# Trimeric Tau Is Toxic to Human Neuronal Cells at Low Nanomolar Concentrations

**DOI:** 10.1155/2013/260787

**Published:** 2013-09-17

**Authors:** Huilai Tian, Eliot Davidowitz, Patricia Lopez, Sharareh Emadi, James Moe, Michael Sierks

**Affiliations:** ^1^Department of Chemical Engineering, Arizona State University, P. O. Box 876106, Tempe, AZ 85287-6106, USA; ^2^Oligomerix, Inc., 3960 Broadway, New York, NY 10032, USA

## Abstract

In Alzheimer's disease (AD), tau aggregates into fibrils and higher order neurofibrillary tangles, a key histopathological feature of AD. However, soluble oligomeric tau species may play a more critical role in AD progression since these tau species correlate better with neuronal loss and cognitive dysfunction. Recent studies show that extracellular oligomeric tau can inhibit memory formation and synaptic function and also transmit pathology to neighboring neurons. However, the specific forms of oligomeric tau involved in toxicity are still unknown. Here, we used two splice variants of recombinant human tau and generated monomeric, dimeric, and trimeric fractions of each isoform. The composition of each fraction was verified chromatographically and also by atomic force microscopy. The toxicity of each fraction toward both human neuroblastoma cells and cholinergic-like neurons was assessed. Trimeric, but not monomeric or dimeric, tau oligomers of both splice variants were neurotoxic at low nanomolar concentrations. Further characterization of tau oligomer species with disease-specific modifications and morphologies is necessary to identify the best targets for the development of biomarker and therapeutic development for AD and related tauopathies.

## 1. Introduction

Alzheimer's disease (AD) is the most common form of dementia, characterized by progressive cognitive impairment, cerebral atrophy, and neuronal loss, with death generally occurring four to eight years after diagnosis [1]. Two pathological hallmarks of AD, extracellular neuritic plaques primarily composed of amyloid beta (A*β*) and intracellular neurofibrillary tangles (NFTs) primarily composed of tau protein, were originally identified in 1907 by Dr. Alois Alzheimer [2]. While great strides have been made in understanding the mechanisms that promote aggregation of A*β* and tau into the hallmark plaques and tangles, comparatively little progress has been achieved in halting or curing the disease. Analysis of familial AD cases implicated production of A*β* as a primary factor in progression of AD, leading to the rise of the amyloid cascade hypothesis which states that A*β* misfolding and aggregation initiates AD pathogenesis and triggers other effects such as tau phosphorylation, aggregation, and tangle formation [3]. The amyloid hypothesis had dominated the field for more than a decade and has driven numerous clinical studies for therapeutic interventions including several immunization studies targeting A*β* [4–6]. However failure of several clinical trials targeting A*β* has cast doubt on its relevance as a therapeutic target [7]. Increasing evidence indicates that tau also plays an important role in the progression of AD. Tau misfolding and aggregation can take place independently of amyloid formation [8], and in many cases the presence of tau lesions is associated with AD without presence of A*β* aggregates [9]. Clearance of A*β* plaques without reducing soluble tau levels is insufficient to ameliorate cognitive decline in double transgenic mice overexpressing A*β* and tau P301L [10]. These results among many others indicate that oligomeric tau may be an important therapeutic target for AD.

**Figure 1:**
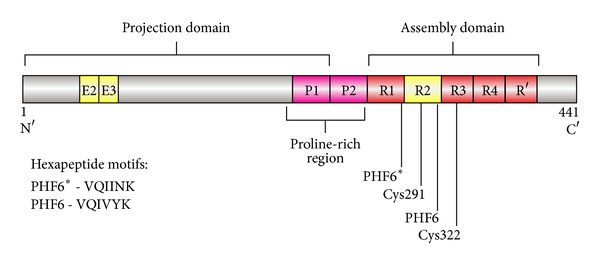
Tau protein structural features in linear diagram. A full-length tau protein with 441 amino acids (tau441 or tau 2N4R) is shown. Alternative splicing showed in yellow rectangles results in a total of six isoforms, denoted by either their total number of amino acids or the number of N′-terminal exons (Ns) and microtubule-associated repeats (Rs).

**Figure 2:**
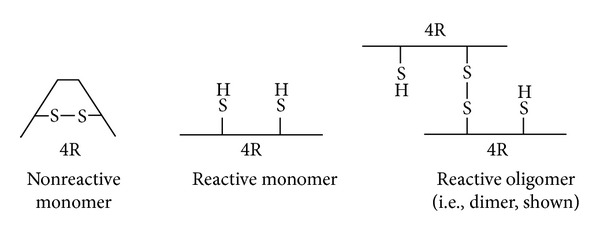
Schematic of nonreactive monomer, reactive monomer, and reactive oligomer. Reactivity implies the ability to form an intermolecular disulfide linkage. Intramolecular disulfide linkage causes formation of nonreactive tau monomer. The free thiols in a reactive monomer allow formation of an intermolecular or intramolecular disulfide linkage. Reactive oligomer has one or more free thiols readily forming disulfide linkage with reactive monomeric tau for the oligomer extension purpose.

Tau in its monomeric form is a microtubule-associated protein crucial for microtubule assembly [11, 12] and stabilization [13]. Six major tau isoforms can be generated by alternative posttranscriptional splicing of exon 2 and exon 3 on the N-terminal projection domain and of exon 10 (Repeat 2) on the assembly domain (Figure 1). Tau contains three or four similar repeats in the microtubule-binding domain (MBD) that binds to and helps promote microtubule stability and function. For example, Repeat 2 and Repeat 3 contain hexapeptide motifs of PHF6* and PHF6, respectively (Figure 1). These motifs increase the tendency to form *β*-sheet structures that can interact with tubulins to form microtubules and also facilitate self-assembly to generate oligomeric and higher-order aggregates [14, 15]. Tau isoforms with or without the second microtubule-binding repeat can aggregate, but only the isoforms with the second repeat can form extended oligomeric forms mediated by disulfide linkages due to the additional cysteine in the second repeat (Figures 1 and 2). Therefore, in this study we utilized tau isoforms containing the second repeat unit to study the role of tau aggregation in neurotoxicity.

Hyperphosphorylation of tau is required for the release of tau from microtubules and its mislocalization to the somatodendritic compartment enabling tau to self-associate into oligomers and higher-order aggregates. However, the hyperphosphorylation of tau is not directly related to its toxicity but rather a mechanism to regulate its interaction with tubulin to stabilize microtubules and to regulate transport along microtubules. Expression of exogenous tau in mature hippocampal neurons leads to blockage of transport along microtubules and degeneration of synapses that can be rescued by phosphorylation of tau by kinase MARK2 to unblock the microtubule tracks [16]. Significantly, tau in the extracellular space is reported to be less phosphorylated than intracellular tau [17, 18] and more toxic in its dephosphorylated state [17]. Extracellular oligomers of recombinant full-length human tau protein were shown to be neurotoxic in mice and impair memory consolidation [19], and similar work at other labs has shown similar effects with recombinant tau oligomers and tau oligomers composed of hyperphosphorylated tau from AD brain. Thus, the hyperphosphorylation of tau associated with disease may be a causal factor in tau self-association into oligomers, but the hyperphosphorylation of tau in and of itself may not be the basis for the toxicity of extracellular tau oligomers. 

Neurofibrillary tangles (NFTs) have traditionally been correlated with neuronal loss [20] and considered to be key intracellular indicators of AD. Approaches for targeting tau aggregation have focused on inhibiting hyperphosphorylation and fibril formation, reducing total tau levels, or stabilizing microtubules [21]. However, accumulating evidence suggests that soluble oligomeric rather than insoluble fibrillar tau species are neurotoxic and play an important role in the onset and progression of AD [21–24]. Although NFTs are a hallmark feature of AD, they can exist in AD neurons for up to 20 to 30 years [25] before postmortem confirmation and therefore are less likely to induce immediate toxicity in AD brain [26]. In animal models of tauopathy, the presence of NFTs does not correlate well with neuronal loss and memory deficits [27]. Reduction in neuronal loss and improvement in memory performance are observed despite an increase in NFTs [28]. In addition, the presence of NFT pathology does not localize well with areas of neuronal loss [29–31], synapse loss or dysfunction in the hippocampus along with microglial activation occurs well before the presence of NFTs [32]. In contrast, oligomeric tau was implicated in numerous studies as playing a key role in AD progression [33–35] and to be a primary initiator of neurotoxicity and neurodegeneration [36]. Oligomeric tau has been identified in early stages of neuronal cytopathology in AD and closely correlates with hyperphosphorylation on microtubule-binding sites [24]. Tau oligomers can propagate endogenous tau pathology throughout the brain similarly to prions, demonstrating their neuronal toxicity [37]. The presence and concentrations of two tau oligomers (140 kDa and 170 kDa) correlate with memory loss in various age rTg4510 mice [33]. Oligomeric tau also induces synaptic and mitochondrial dysfunction [19]. Although tau is predominantly intracellular, the role of extracellular tau is gaining attention as extracellular oligomeric tau can have acute effects on long-term potentiation in hippocampal slices and can transmit pathology to healthy neurons [37]. Detection of oligomeric tau levels in human CSF and blood is also a promising AD diagnostic biomarkers along with total and hyperphosphorylated tau levels [38]. Because of the important role of oligomeric tau in AD and the recognition of the importance of extracellular tau in disease, it is critical to identify the key toxic tau species in disease etiology. Here we show our studies of the extracellular neurotoxicity of monomeric, dimeric, and trimeric forms of two four-repeat recombinant human tau variants to help identify the key tau species involved in the onset and progression of AD.

## 2. Material and Methods

### 2.1. Recombinant Human Tau (rhTau) Preparation and Purification

rhTau was purified as monomers from bacterial (BL21 DE3) clones with tau constructs in the pET21B and pET29a vectors. Standard methods were used to grow and induce the protein with 1 mM IPTG. Pelleted cells were lysed with CelLytic B lysis buffer, lysozyme, benzonase, and protease inhibitors according to the manufacturer's protocol (Sigma Aldrich, St. Louis, MO). Cation exchange (GE Healthcare Life Sciences) was used for the first step of purification with SP-Sepharose resin for both tau constructs, and 300 mM NaCl in 25 mM Tris-HCl pH 7.4 was used to elute tau protein. Amicon Ultra Centrifugal Devices (Millipore) were used to buffer-exchange the protein preparations into 50 mM Tris-HCl pH 7.4. Protein concentration was determined using a BCA assay (Thermo Fisher Scientific). Tau oligomers were generated by incubating tau monomers at a concentration of 5 *μ*M in 50 mM Tris buffer pH 7.4 with 100 mM NaCl at 37°C overnight. The monomeric and oligomeric species were resolved by 6% PAGE, eluted, and buffer-exchanged into 50 mM Tris-HCl. Fractions were analyzed by nonreducing SDS-PAGE to minimize degradation of oligomeric proteins and silver staining to enhance the signal and to verify the purity of tau variants. Protein concentration was determined using the BCA assay. 

### 2.2. Height Distribution Analysis

 AFM sample preparation and imaging were performed as described previously [39–44]. Aliquots of 10 *µ*L 0.50 *µ*M purified tau variants in 50 mM Tris-HCl buffer were deposited on separate mica pieces for imaging using MultiMode AFM Nanoscope IIIA system (Veeco/Digital instruments, Santa Barbara, CA) which was set in tapping mode and equipped with silicon AFM probes (VISTA probes, Nanoscience Instruments). Height distribution analysis of the different tau samples was fit to a normal distribution probability model using Gwyddion 2.20. All detectable protein molecules were assumed to be spherical and the height values approximate their diameters. 

### 2.3. Cell Culture and Treatments

SH-SY5Y human neuroblastoma cell lines (American Tissue Culture Collection) were cultivated in tissue culture flask (Falcon by Becton Dickinson Labware). Cells were grown in a medium containing 44% v/v Ham's F-12 (IrvineScientific), 44% v/v MEM Earle's salts (IrvineScientific), 10% v/v denatured fetal bovine serum (FBS) (Sigma Aldrich), 1% v/v MEM nonessential amino acids (Invitrogen), and 1% v/v antibiotic/antimycotic (Invitrogen). Media were renewed once every two to three days. The cells were passaged to a new flask when they were confluent in the flask. For toxicity studies, the SH-SY5Y cells were seeded in a 48-well cell culture cluster plate (Costar by Corning Incorporated) with 5 × 10^4^ cells/well in 300 *μ*L fresh medium. Each experiment was conducted in triplicate. Cell density was estimated by reading a fixed volume on a hemocytometer. After growth in a 37°C incubator for 24 hours, the tissue culture media were replaced with fresh serum-free media for the neurotoxicity test on nondifferentiated cells. To investigate tau toxicity on cholinergic neurons, a duplicate set of the cultured cells was induced into cholinergic-like phenotype by incubation with retinoic acid at a final concentration of 10 *μ*M for 3 to 5 days [43, 45–47]. The cultivated nondifferentiated and cholinergic-like neurons were treated with monomeric, dimeric, and trimeric variants of 1N4R and 2N4R at final concentrations of 2.26 nM, 4.50 nM, 11.15 nM, and 15.50 nM. A PBS negative control was used as a standard for subsequent LDH assay analysis. Cultures were incubated with tau species at 37°C and sampled at 3, 18, 24, and 48 hour time points by harvesting 30 *μ*L/well aliquots of culture supernatant. 

### 2.4. LDH Assay

The LDH protocol is adapted from a commercial kit (Sigma Aldrich) based on the generic protocol of Decker and Lohmann-Matthes [48]. The LDH assay was performed as described previously [40]. Absorbance was measured at 490 nm (reference wavelength 690 nm). Relative absorbance values were calculated by subtracting the reference values from the values obtained at 490 nm. LDH% values greater than 150 are considered toxic.

### 2.5. Statistical Analysis

The relative absorbance values of all samples were normalized to those of controls which were set as 100% for each independent experiment. Group mean values were analyzed by one-way ANOVA with *P* < 0.05 standard and LSD post hoc significant differences test. All analyses were performed with SPSS 21.0 (IBM Corp., Armonk, NY).

## 3. Results 

### 3.1. rhTau Aggregate Analysis

**Figure 3:**
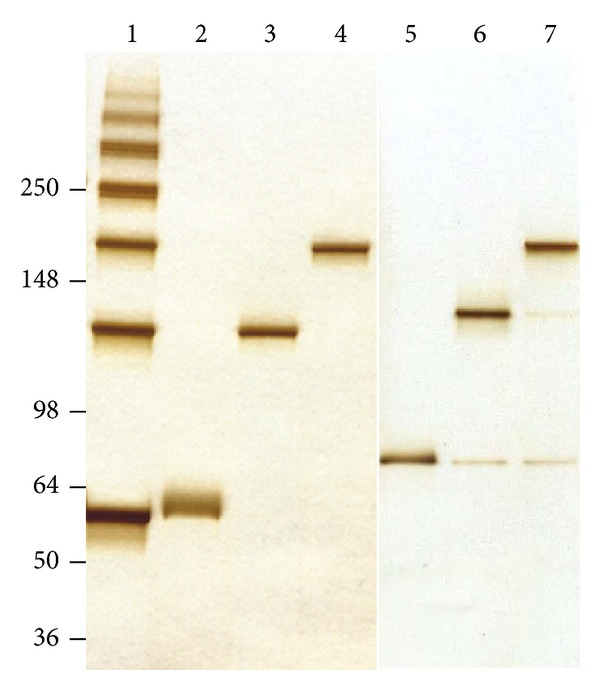
Recombinant human tau (rhTau) monomeric and oligomeric species production and purification. rhTau 1N4R enriched with disulfide-mediated tau oligomers (lane 1) was used for the purification of monomeric, dimeric, and trimeric tau species (lanes 2–4), 2N4R purified monomeric, dimeric, and trimeric species (lanes 5–7).

**Figure 4:**
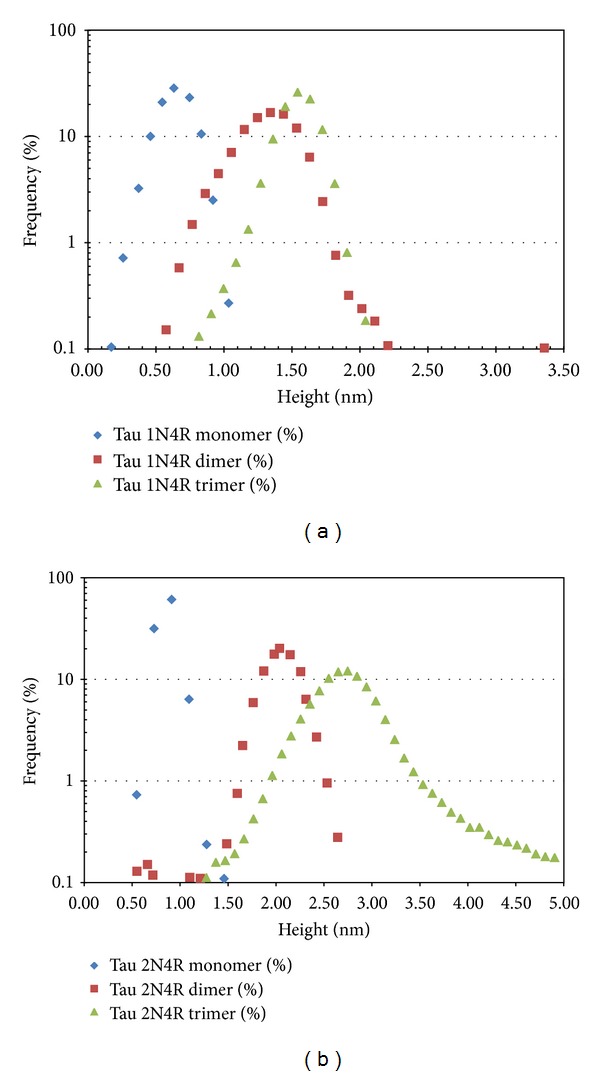
Plots of height distribution of monomeric, dimeric, and trimeric fractions of rhTau 1N4R (a) and tau 2N4R (b). The height value of each particle was measured using Gwyddion. The numbers of particles falling in continuous size ranges were calculated and normalized into count percentages. The peak values give an approximate value for each tau species particle size. As expected, high-degree oligomers are larger than low-degree oligomers within the same isoform, and corresponding oligomeric aggregates from the longer isoform are larger than aggregates from the shorter isoform.

We expressed recombinant human tau in a bacterial host system to eliminate any posttranslational phosphorylation of tau and therefore remove any potential effects that phosphorylation may have on tau aggregation or loss of function. The resulting nonphosphorylated human recombinant tau (NPrhTau) monomers contain reactive cysteine groups with free thiols, facilitating the formation of intramolecular disulfide bonds to make stable nonreactive monomers and the formation of intermolecular disulfide bonds to produce tau oligomers and higher-degree aggregates (Figure 2). The polymerization reaction is controlled by incubation time and protein concentration. The nonreactive monomeric, dimeric, and trimeric forms of both the 2N4R and 1N4R splice variants generate stable aggregate morphologies with defined size profiles dependent on the degree of oligomerization and length of the splice variant as evidenced by SDS-PAGE (Figure 3) and AFM height distribution analysis (Figure 4). The oligomer heights increment for each additional monomeric tau unit is fixed within a certain isoform, which is 0.5 nm for 1N4R variants and 1.0 nm for the 2N4R variants (Figure 4). The size of each respective 2N4R species is also larger than the corresponding 1N4R species (Figures 3 and 4) as expected given that tau 2N4R contains the extra N-terminal insert compared with the 1N4R variants. 

### 3.2. Extracellular rhTau Induced Neurotoxicity Test

**Figure 5:**
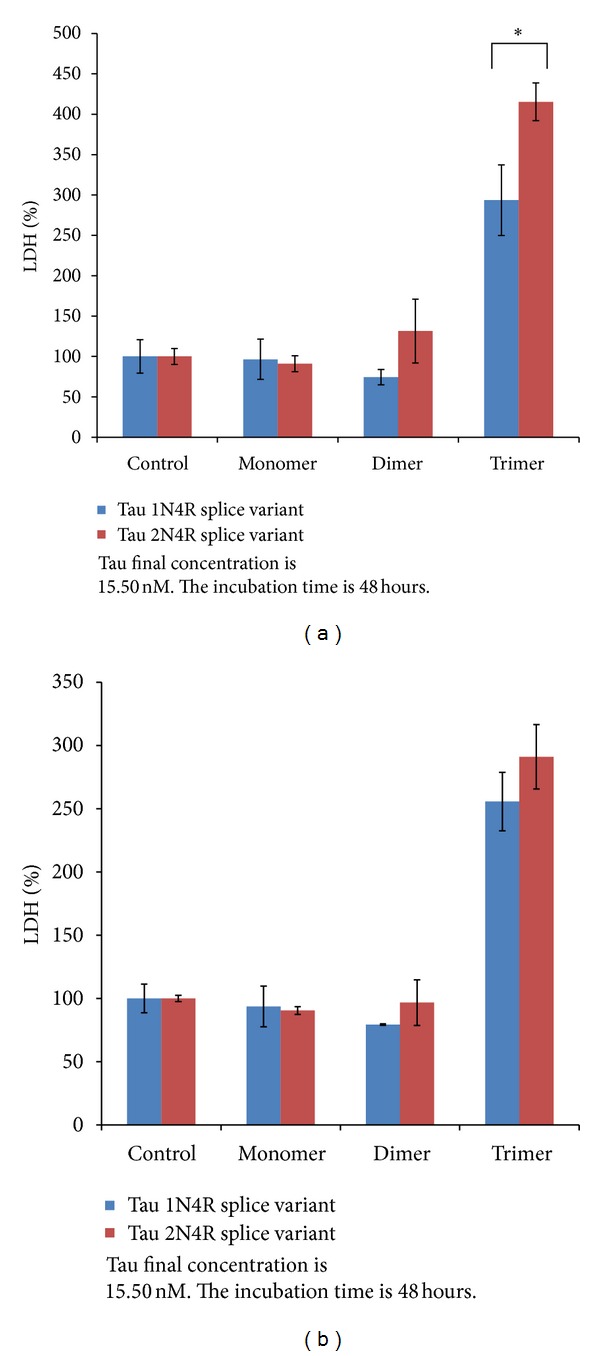
Neurotoxicity of extracellular 15.5 nM monomeric, dimeric, and trimeric forms of 1N4R and 2N4R tau variants toward (a) nondifferentiated human neuroblastoma cells (SH-SY5Y) and (b) Retinoic-acid-differentiated SH-SY5Y cells was measured after 48-hour incubation using an LDH assay. For both four-repeat tau isoforms, trimeric form is more neurotoxic than monomeric and dimeric forms (*P* < 0.001) on either neuron type. Full-length trimeric rhTau is more neurotoxic than 1N4R trimeric rhTau. (*P* < 0.05).

**Figure 6:**
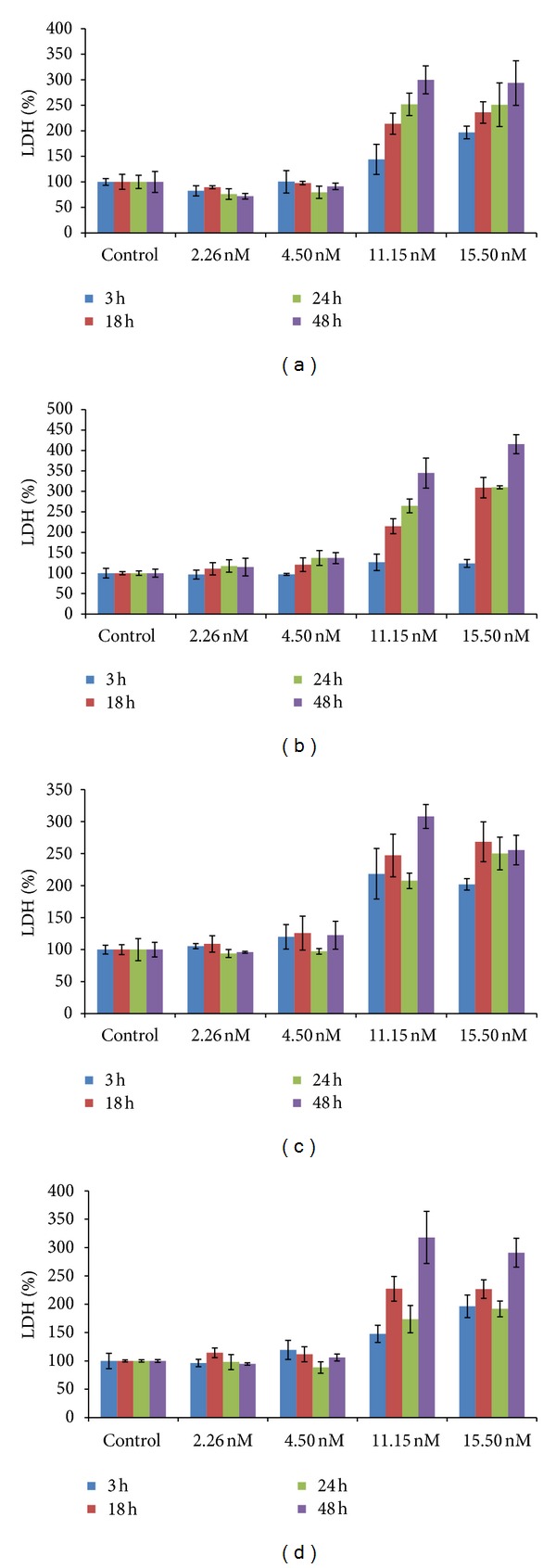
Time and concentration dependence of neurotoxicity induced by trimeric rhTau (1N4R and 2N4R) toward neuroblastoma cells measured by LDH assay. Nondifferentiated SH-SY5Y cells incubated with (a) 1N4R tau and (b) 2N4R tau; retinoic-acid-differentiated SH-SY5Y cells incubated with (c) 1N4R tau and (d) 2N4R tau.

**Figure 7:**
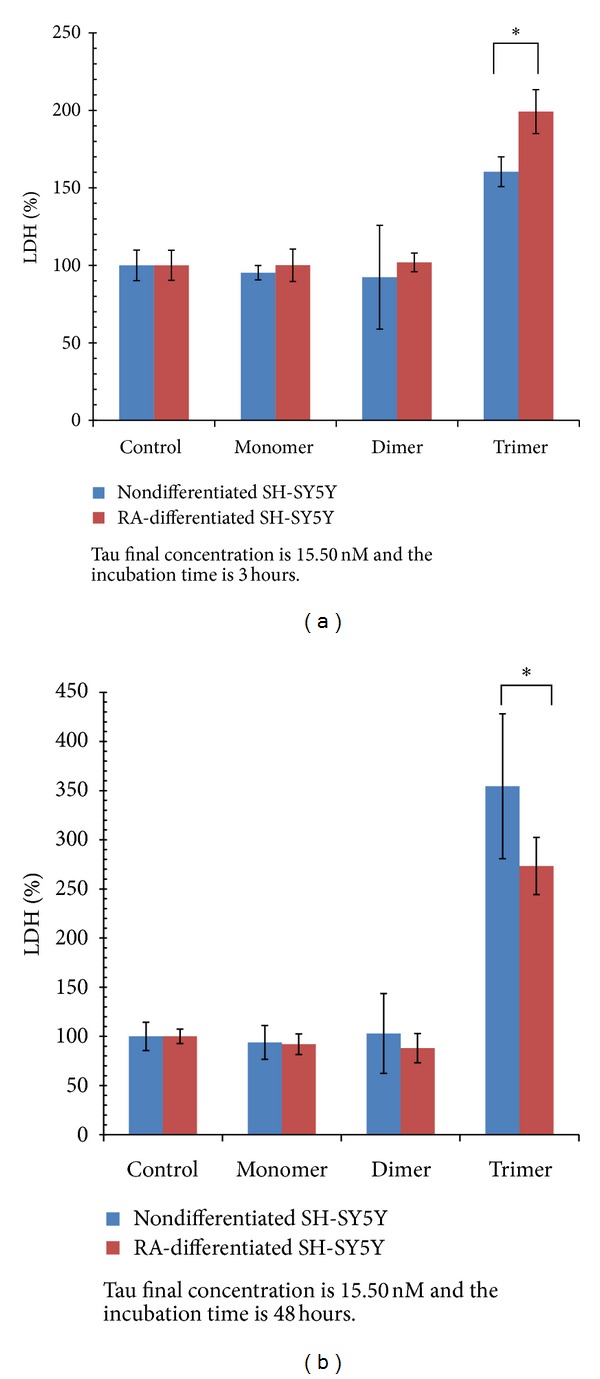
Comparison of rhTauinduced neurotoxicity toward nondifferentiated SH-SY5Y cells and retinoic-acid- (RA-) differentiated SH-SY5Y cells. The data combine toxicity results of 15.5 nM monomeric, dimeric, and trimeric forms of both 1N4R and 2N4R tau variants. (a) After 3 hours-incubation, RA-differentiated SH-SY5Y cells are more vulnerable to extracellular trimeric rhTau toxicity than nondifferentiated SHSY-5Y cells are (*P* < 0.05). (b) After 48-hours incubation, nondifferentiated SH-SY5Y cells are more vulnerable to extracellular trimeric rhTau toxicity than RA-differentiated SH-SY5Y cells (*P* < 0.05).

While neither the monomeric or dimeric forms of tau from either the 1N or 2N splice variants displayed detectable toxicity, the trimeric form of both variants exerted marked toxicity toward nondifferentiated (Figure 5(a)) and retinoic acid induced cholinergic-like neurons (Figure 5(b)) with LDH values well above the toxic threshold of 150 at low nanomolar concentrations (11.15 nM, and 15.50 nM). The full-length 2N4R trimeric tau form displayed significantly higher toxicity than the 1N4R trimeric form toward nondifferentiated neurons (Figure 5(a)), although the effect is diminished in the cholinergic-like neurons (Figure 5(b)). When trimeric tau was added to nondifferentiated SH-SY5Y cells, an increase in toxicity was observed with time at the highest concentrations for both the 1N4R (Figure 6(a)), and 2N4R (Figure 6(b)) trimeric variants. However, when trimeric tau was added to the cholinergic-like neurons, the toxicity of the 1N (Figure 6(c)) and 2N (Figure 6(d)) variants was relatively consistent over the first 24 hours, but increased after 48 hours. Both variants of trimeric tau showed increased toxicity toward the cholinergic-like neurons compared to the nondifferentiated neurons at short incubation times (Figure 7(a)) but the reverse was observed at longer incubation times (Figure 7(b)). 

## 4. Discussion 

While the amyloid cascade hypothesis [49] has dominated studies into the etiology of AD over the last decade or more, the importance of tau in the onset and progression of AD is steadily becoming more apparent. Tau pathology has been observed in the absence of A*β* deposits in children and young adult cases, and tau aggregates in the entorhinal-hippocampal regions precede the onset of A*β* pathology [8, 9]. Numerous studies have shown that various oligomeric forms of A*β* are toxic to neurons and can impair cognitive performance [50, 51], thus implicating their potential role as valuable biomarkers for diagnosing AD [42, 52, 53]. Similar to the important role of various soluble oligomeric A*β* species in AD, different soluble oligomeric forms of tau may also play a critical role in AD, also causing neuronal loss and cognitive dysfunction [19, 54, 55]. Therefore to facilitate diagnoses and therapeutic treatments for AD, it is important to identify the key tau species involved in the onset and progression of the disease. Given that tau has multiple splice variants and posttranslational modification sites, we attempted to simplify the complex diversity of tau forms by focusing on two nonphosphorylated human recombinant tau isoforms, 1N4R and 2N4R. These two four-repeat (4R) isoforms of tau both have all four repeats of the microtubule-associated domains and are more prone to form the aggregates readily phosphorylated by brain protein kinases than those with only three repeats (3R) [56] due to the presence of Repeat 2 with a microtubule-affinity enhancing hexapeptide motif [14, 15] and an additional cysteine that forms disulfide linkages to stabilize the aggregates.

The most disease-relevant tau material to use to study toxicity of extracellular tau forms would be well characterized tau oligomers purified from AD cerebrospinal fluid (CSF) using methods to preserve their posttranslational modifications, including phosphorylation, glycation, ubiquitination, aggregation, and truncation. Preparations from several non-AD and AD cases would be necessary to understand the significance of the results. Here we performed an initial study focused specifically on unmodified tau protein oligomers and control monomer to specifically understand the relevance of oligomer structure to extracellular toxicity.

We determined the toxicity of the different tau variants using both nondifferentiated and cholinergic-like neuroblastoma cell lines to determine how aggregate size and cell phenotype affected toxicity. Cholinergic cells are particularly vulnerable in AD with significant neuronal loss in the nucleus basalis of Meynert (NBM), that is, the hippocampus and the cortex [57]. NBM is enriched in cholinergic cells and undergoes degeneration and a significant decrease of acetylcholine production in AD [58]. Decreased levels of acetylcholine and a number of other cortical cholinergic markers lead to clinical dementia and impairment in cognitive function [58], indicating that cholinergic cells are particularly vulnerable in AD. Here we show that trimeric, but not monomeric or dimeric, tau is toxic to neuronal cells at low nanomolar concentrations and that the full-length 2N tau variant is more toxic than the shorter 1N variant to nondifferentiated neurons (Figure 5). Both trimeric tau variants cause toxicity to both nondifferentiated SH-SY5Y cells and retinoic acid induced cholinergic-like neurons when tau was applied extracellularly at nanomolar levels (Figure 6). However, the cultured cholinergic-like neurons show increased susceptibility to trimeric tau induced toxicity at short incubation times compared with similar nondifferentiated neurons (Figure 7(a)), perhaps partially accounting for the increased vulnerability of cholinergic-like neurons in AD. Since the nondifferentiated cells were equally susceptible to trimeric tau induced toxicity at longer incubation times (Figure 7(b)), these results suggest that toxicity of extracellular trimeric tau is not dependent on receptors or proteins specifically associated with cholinergic cells but that toxicity might be facilitated by them. Our results are consistent with a recent study showing that low molecular weight (LMW) misfolded tau species exclusive of monomeric tau can be endocytosed by neurons and transported both anterogradely and retrogradely to induce endogenous tau pathology in vivo while fibrillar tau and brain-derived filamentous tau cannot be endocytosed [59]. This suggests that tau toxicity may be spread through cells in certain brain regions by endocytosis of trimeric and larger oligomeric forms of tau and that this uptake is facilitated in cholinergic neurons. 

Neuronal toxicity of oligomeric tau may share similar properties to that of oligomeric A*β* where the critical feature involved in neuronal toxicity is the aggregation state of the protein more than posttranslational modifications [23, 60]. While there are a wide variety of tau variants that occur in vivo including different posttranslational modifications, splice variants, and aggregated species, this study begins to more systematically probe the role of selected tau variants in AD. Further studies are needed to determine the contribution of splice variants and AD-specific posttranslational modifications found in extracellular tau to the toxicity of the tau variants and to how these tau variants affect other neuronal models including primary neurons or induced pluripotent stem cells. Well characterized reagents that can selectively identify specific tau variants and morphologies will be useful for these further studies.
